# Epimutations in both the *TESK2* and *MMACHC* promoters in the Epi-*cblC* inherited disorder of intracellular metabolism of vitamin B_12_

**DOI:** 10.1186/s13148-022-01271-1

**Published:** 2022-04-19

**Authors:** Abderrahim Oussalah, Youssef Siblini, Sébastien Hergalant, Céline Chéry, Pierre Rouyer, Catia Cavicchi, Renzo Guerrini, Pierre-Emmanuel Morange, David Trégouët, Mihaela Pupavac, David Watkins, Tomi Pastinen, Wendy K. Chung, Can Ficicioglu, François Feillet, D. Sean Froese, Matthias R. Baumgartner, Jean-François Benoist, Jacek Majewski, Amelia Morrone, David S. Rosenblatt, Jean-Louis Guéant

**Affiliations:** 1 INSERM UMR_S 1256, Nutrition, Genetics, and Environmental Risk Exposure (NGERE), Faculty of Medicine of Nancy, University of Lorraine, 9 Avenue de la Forêt de Haye, 54000 Nancy, France; 2 Reference Center for Inborn Errors of Metabolism (ORPHA67872), University Hospital of Nancy, 54000 Nancy, France; 3 Department of Molecular Medicine, Division of Biochemistry, Molecular Biology and Nutrition, University Hospital of Nancy, 54000 Nancy, France; 4 Molecular and Cell Biology Laboratory of Neurometabolic Diseases, Paediatric Neurology Unit and Laboratories, Meyer Children’s Hospital, Viale Pieraccini 24, 50139 Florence, Italy; 5 Department of NEUROFARBA, University of Florence, Florence, Italy; 6 INSERM UMR_S 1263, Center for CardioVascular and Nutrition Research (C2VN), Aix-Marseille University, 13385 Marseille, France; 7 INSERM, BPH, U1219, Université Bordeaux, 33000 Bordeaux, France; 8 Department of Human Genetics, McGill University and Research Institute, McGill University Health Centre, Montreal, QC H4A 3J1 Canada; 9 Departments of Pediatrics and Medicine, Columbia University, New York, USA; 10 Children’s Hospital of Philadelphia, Perelman School of Medicine at the University of Pennsylvania, Philadelphia, PA USA; 11 Division of Metabolism, University Children’s Hospital, University of Zürich, Zürich, Switzerland; 12 Biochemistry Hormonology Laboratory, Robert-Debré University Hospital, APHP, 48 bd Serurier, 75019 Paris, France; 13 Department of Hepato-Gastroenterology, University Hospital of Nancy, 54000, Nancy, France

**Keywords:** Epi-cblC, Secondary epimutation, Promoter hypermethylation, *MMACHC*, *TESK2*, Methylmalonic aciduria and homocystinuria, *cblC* type

## Abstract

**Background:**

epi-*cblC* is a recently discovered inherited disorder of intracellular vitamin B_12_ metabolism associating hematological, neurological, and cardiometabolic outcomes. It is produced by an epimutation at the promoter common to *CCDC163P* and *MMACHC*, which results from an aberrant antisense transcription due to splicing mutations in the antisense *PRDX1* gene neighboring *MMACHC*. We studied whether the aberrant transcription produced a second epimutation by encompassing the CpG island of the *TESK2* gene neighboring *CCDC163P*.

**Methods:**

We unraveled the methylome architecture of the *CCDC163P*–*MMACHC* CpG island (CpG:33) and the *TESK2* CpG island (CpG:51) of 17 epi-*cblC* cases. We performed an integrative analysis of the DNA methylome profiling, transcriptome reconstruction of RNA-sequencing (RNA-seq), chromatin immunoprecipitation sequencing (ChIP-Seq) of histone H3, and transcription expression of *MMACHC* and *TESK2*.

**Results:**

The *PRDX1* splice mutations and activation of numerous cryptic splice sites produced antisense readthrough transcripts encompassing the bidirectional *MMACHC*/*CCDC163P* promoter and the *TESK2* promoter, resulting in the silencing of both the *MMACHC* and *TESK2* genes through the deposition of SETD2-dependent H3K36me3 marks and the generation of epimutations in the CpG islands of the two promoters.

**Conclusions:**

The antisense readthrough transcription of the mutated *PRDX1* produces an epigenetic silencing of *MMACHC* and *TESK2*. We propose using the term 'epi-digenism' to define this epigenetic disorder that affects two genes. Epi-*cblC* is an entity that differs from *cblC.* Indeed, the *PRDX1 and TESK2* altered expressions are observed in epi-*cblC* but not in *cblC*, suggesting further evaluating the potential consequences on cancer risk and spermatogenesis.

**Supplementary Information:**

The online version contains supplementary material available at 10.1186/s13148-022-01271-1.

## Introduction

Epigenetic regulation of gene expression through DNA methylation is a source of genetic variation and can induce transcriptional haploinsufficiency [1]. Epigenetic diseases can be caused by aberrant DNA methylation marks, also called epimutations, which represent an underlying molecular mechanism of disease. In the setting of inherited metabolic disorders, we have previously reported a new type of inherited defect of vitamin B_12_ (cobalamin, cbl) metabolism with an epimutation in the promoter of the *MMACHC* gene, which we called epi-*cblC* [2]. Patients with epi-*cblC* have the same clinical presentation as *cblC* type, an autosomal recessive inherited disorder of intracellular vitamin B_12_ metabolism with combined methylmalonic aciduria and homocystinuria (OMIM phenotype ID: 277400) due to mutations in the *MMACHC* gene (metabolism of cobalamin-associated C; OMIM gene ID, 609831). In contrast, epi-*cblC* cases harbor an epimutation with an aberrant methylation of the CpG island (CpG:33) in the *MMACHC* promoter that silences the expression of the *MMACHC* gene. *MMACHC* belongs to a gene trio in which it is a sense gene flanked by *CCDC163P* and *PRDX1* in the opposite orientation (trio with antisense (reverse, R1)/sense (forward, F2)/antisense (reverse, R3)) [2]. We found mutations in *PRDX1* that produced an antisense transcript encompassing the *MMACHC/CCDC163P* bidirectional promoter, resulting in an H3K36me3 mark and the generation of the epimutation [2]. The splice acceptor variants induced readthrough transcripts extending beyond the normal poly(A) addition site, thus skipping the transcription termination signal of *PRDX1* [2]. Most epi-*cblC* patients had a combination of alleles*,* with an epimutation in one allele and an *MMACHC* pathogenic genetic variant carried in *trans* [2]. One patient had a bi-allelic *MMACHC* epimutation due to the homozygous *PRDX1*:c.515-1G > T variant transmitted by both parents. In this homozygous case, we found that the bi-allelic epimutation produces the complete silencing of *MMACHC* in the patient’s fibroblasts [2]. No *PRDX1* variant was detected in one of two *epi-cblC* cases reported in China, suggesting that the epimutation could also be triggered by a non-genetic mechanism [2]. Importantly, we demonstrated the presence of the *MMACHC* secondary epimutation in DNA from sperm, showing that the epimutation escaped erasure in spermatozoa [2]. This observation may be explained by the ubiquitously high expression of *PRDX1* in germ cells [2].

The aberrant antisense transcript of *PRDX1* encompasses the *CCDC163P*/*MMACHC* bidirectional promoter. *TSK2* is an antisense gene neighboring *CCDC163P*, with the CpG island ‘CpG:51’ in its promoter region. However, whether the aberrant transcription encompasses the CpG island ‘CpG:51’ and produces a *TESK2* epimutation was not considered. To address this hypothesis, we gathered data from the 17 epi-*cblC* patients described from Europe and North America. We unraveled the methylome architecture of the *CCDC163P*/*MMACHC* CpG island (CpG:33) and the *TESK2* CpG island (CpG:51) by studying the epigenome-wide DNA methylation profile of the 17 epi-*cblC* cases [2, 3]. We performed an integrative analysis of the DNA methylome profiling, RNA-sequencing (RNA-seq) with de novo transcriptome reconstruction, reverse transcription-quantitative polymerase chain reaction (RT-qPCR), and chromatin immunoprecipitation sequencing (ChIP-Seq). We revealed epimutations in both the *CCDC163P*/*MMACHC* CpG island (CpG:33) and the *TESK2* CpG island (CpG:51) that produced silencing in both genes.

## Methods

### Overview of the study design

First, we used an EWAS approach to look for epigenomic signatures associated with the epi-*cblC* phenotype. In a second step, we investigated whether the DNA methylation signatures revealed in the EWAS translate in terms of chromatin remodeling (ChIP-seq analysis), gene expression (RNA-seq and RT-qPCR analyses), and transcript structure (splicing analysis). We studied fibroblasts from skin biopsies since most reference centers use these cells for phenotyping inherited disorders of intracellular vitamin B_12_ metabolism.

### DNA methylome study design and patients description

We performed a case–control EWAS to look for significant epigenomic signatures associated with an epi-*cblC* phenotype. The epi-*cblC* phenotype was defined by one of the three following conditions: i) composite *MMACHC* epimutation/*MMACHC* mutation, ii) heterozygous *MMACHC* epimutation, iii) homozygous *MMACHC* epimutation. We studied 17 patients with proven *MMACHC* epimutation, as previously described [2, 3]. All patients exhibited aberrant methylation on the CpG island ‘CpG:33’ on the *CCDC163*–*MMACHC* bidirectional promoter (Chr1: 45,965,587–45,966,049, GRCh37). Of the 17 patients with *MMACHC* epimutation, three were assessed at the Reference Center for Inborn Errors of Metabolism, University Hospital of Nancy, France (CHU-12122, a female patient with composite *MMACHC* epimutation/*MMACHC* mutation (composite epi-*cblC* disease) who died at 1-month of life from cardiometabolic decompensation and two relatives with heterozygous *MMACHC* epimutation [CHU-14061, the father, and CHU-14067, the grandfather]) [2] and three were assessed at the Department of Human Genetics, Montreal, Canada (WG-3838, a female patient with a composite epi-*cblC* disease who died at the age of two months from a sudden cardiac arrest with an acute renal failure, her father [CDH-867] who had a heterozygous *MMACHC* epimutation, and a 59-year-old male patient with a composite epi-*cblC* disease [WG-4152]) [2]. The remaining eleven patients were assessed at the Molecular and Cell Biology Laboratory of Neurometabolic Diseases and Paediatric Neurology Unit and Laboratories, Meyer Children’s Hospital, Florence, Italy. Of the eleven patients, eight had a composite epi-*cblC* disease and three corresponded to a trio composed of a proband exhibiting a homozygous *MMACHC* epimutation and the two parents with heterozygous *MMACHC* epimutation [3]. In total, of the 17 patients analyzed in the study, eleven had a composite *MMACHC* epimutation/*MMACHC* mutation, five had a heterozygous *MMACHC* epimutation, and one had a homozygous *MMACHC* epimutation. For the control group, we used in silico data generated using the Infinium Human Methylation 450 k BeadChip array from the MARTHA cohort, which included 350 unrelated European-ancestry ambulatory subjects recruited in Marseille (France) between January 1994 and October 2005 [4]. All adult patients and proband’s parents assessed at the Reference Center for Inborn Errors of Metabolism at the University Hospital of Nancy gave their informed written consent for performing the analyses, as previously reported [2]. The Columbia family provided informed consent, and the study was approved by the Columbia IRB [2]. Informed consent was obtained from all participants included in the Italian study, as previously reported (Ethics Committee of the Tuscany Region (No. CS_01/2021) [3].

### DNA methylome analysis, quality controls, and statistical analyses

We carried out bisulfite conversion of 600 ng of DNA extracted from whole blood using the EZ DNA Methylation kit (Zymo Research, Proteigene, Saint-Marcel, France). The genome-wide profiling of DNA methylome was determined using the Infinium Human Methylation 450 k BeadChip array (Illumina, Paris, France) of the Infinium MethylationEPIC BeadChip array (Illumina, Paris, France), according to the manufacturer’s instructions. The arrays were scanned on an Illumina iScan® system, and raw methylation data were extracted using Illumina’s Genome Studio methylation module. For each CpG probe, the methylation level was described as a β value, ranging between 0 (fully unmethylated CpG probe) and 1 (fully methylated CpG probe). Background correction and normalization were implemented using the SWAN method (R Package Minfi) [5]. Probe annotation information, including sequence and chromosome location for Infinium Human Methylation 450 k and Infinium MethylationEPIC BeadChip arrays, was retrieved from the Manifest Files. We visually inspected the genome-wide distribution of the CpG probes according to their β value. We performed principal component analysis (PCA) to assess the clustering of methylation profiles according to the whole methylation landscape of the analyzed samples. The top ten principal components (eigenvectors, EV) were calculated with their respective eigenvalue. PCA plots were used to report on the three top eigenvalues. We compared the mean β values of each CpG probe between the two subgroups using the *t* test with Bonferroni correction. Output data included the mean β values in each subgroup, the difference of β values, the nominal *P*-value, and the Bonferroni corrected *P*-value. We used the smoothed *P*-value transformation by converting nominal *P*-values obtained from the *t* test to smoothed *P*-values using a window radius of 3, as previously reported [2, 3, 6]. All statistical analyses were performed using the SNP & Variation Suite (v8.8.1; Golden Helix, Inc., Bozeman, MT, USA) and MedCalc, version 19.5.3 (MedCalc Software, Ostend, Belgium).

### Whole-genome chromatin Immunoprecipitation sequencing

ChIP-Seq was performed on patient’s fibroblasts and control fibroblast lines without inherited metabolic defects in the cobalamin pathway, as previously reported [2]. According to the manufacturer's instructions, the immunoprecipitation and reverse crosslinking were performed with the IP-Star SX-8G (Diagenode) using the Auto-Histone ChIP kit (Diagenode). ChIP-Seq libraries were prepared using the Kappa Library Preparation Kit Illumina Platforms (Kappa Biosystems) and Illumina TruSeq adapters (Illumina) according to the manufacturer’s instructions. Samples were sequenced using the HiSeq 2000 with a selected read length of 50 bp. Duplicated reads were removed using PICARD. Peaks were called using Model-based Analysis of ChIP-Seq MACS2 with input DNA as control and using the broad peak mod [7]. In order to generate Wiggle (WIG) and Tiled Data Format (TDF) files, HOMER (4.7) [8] and Integrative Genomics Viewer (2.3.67) [9] were used. Tags were normalized to 10 million reads to generate tracks for visualization.

### RNA sequencing

As previously described, RNA depleted of rRNA transcripts was extracted from fibroblasts obtained by skin biopsies of CHU-12122 and WG-3838 epi-*cblC* cases, 3 *cblC* cases with the c.270_271insA mutation, 3 *cblG* cases (another inherited defect of vitamin B_12_ intracellular metabolism due to mutations in methionine synthase (*MTR*) gene) and 4 control fibroblasts using RiboMinus™ (Thermo Scientific, Villebon, France) [2]. cDNA libraries were prepared using the TruSeqTM RNA Sample Preparation Kit (Illumina, San Diego, CA, USA). RNA sequencing was performed using the Illumina Hi-scan sequencer. Reads were classified according to known 5′ and 3′ boundaries of annotated genes. RNA-sequencing FASTQ files for the two composite epi-*cblC* samples compiled 150-nucleotide-long paired-end reads. The control group originated from a previous study [10]. All samples were regrouped and analyzed using the same bioinformatics pipeline, with quality control, adapter removal, read alignment, and manipulation, as previously described [11]. The alignment step was performed on the human reference genome GRCh38, with two splice-aware tools, HISAT v2.0.4 [12] and STAR v2.5.1b [13], that accounted for splicing junctions and genetic polymorphisms. From this point on, as we observed and hypothesized that the RNA splicing mechanisms were abnormal in the genomic region spanning from *TESK2* to *PRDX1*, we could not rely on reference-based transcriptome reconstruction methods, which would not efficiently report on the real isoform population and abundances in this region. We extracted the high-quality reads aligning inside the genomic interval chr1:45,443,800–45,543,000 (*TESK2* to *PRDX1*) and performed de novo transcriptome assembly with Trinity v2.6.5 [14]. The Trinity pipeline was launched with default parameters for paired-end and unstranded reads, with the ‘–jacard_clip’ flag to account for high gene density, with possible overlapping at these genomic coordinates. Besides the two composite epi-*cblC* samples, 4 controls were suitable for the analysis and were used as controls against the composite epi-*cblC* cases. All samples presented with enough coverage in high-quality reads in the region of interest (> 10,000) to achieve transcript reconstruction, providing enough read depth for unbiased estimation of their expression levels. Reconstructed transcripts were then aligned with blastn (with parameters ‘-evalue 1e-10’ and ‘-max_target_seqs 3’) against a cDNA database obtained with BioMart (https://www.ensembl.org/biomart/) and comprising all known isoforms of the four genes *TESK2*, *CCDC163*, *MMACHC,* and *PRDX1*. Criteria for a successful transcript assignment to a known gene product of interest were as follows: i) biotype = protein-coding; ii) select the highest alignment length among candidates; iii) subject length = alignment length and is > 300 nt; iv) a number of mismatches allowed < 4; and v) a maximum number of gaps = 1. Finally, all transcripts were quantified using Kallisto v0.43 [15], which reported the normalized expression levels as estimated read counts and transcript-per-million (TPM) unit.

### Quantification of *MMACHC*, *TESK2*, and *PRDX1* aberrant transcripts mRNA expression

Besides quantification from RNA-seq data, we also quantified the expression of *MMACHC* and *TESK2* transcripts and *PRDX1* aberrant transcript using reverse transcription-quantitative polymerase chain reaction (RT-qPCR). Fibroblasts from an epi-*cblC* case (CHU-12122) and control fibroblasts were cultured in DMEM medium supplemented with 10% v/v heat-inactivated fetal bovine serum, 1% v/v pyruvate, and 1% v/v penicillin–streptomycin. Cells were maintained at 37 °C and in 5% CO_2_. All the reagents were obtained from Sigma-Aldrich. RNA was extracted using NucleoSpin RNA Plus (Macherey–Nagel, Hœrdt, France). After the extraction, a DNase digestion step was carried out to remove DNA contamination using the DNA-free™ DNA Removal kit (Thermo Fisher Scientific France, Illkirch-Graffenstaden, France). RNA was reverse-transcribed using PrimeScript™ RT Master Mix (Takara Bio Europe, Saint-Germain-en-Laye, France); 2 µL of cDNA was used for qPCR with TB Green Premix Ex Taq (Tli RNase H Plus) (Takara Bio Europe, Saint-Germain-en-Laye, France) in a 20 µL reaction volume with the forward and the reverse primers specific for each of the studied genes (Additional file 3: Table S1) at a concentration of 0.2 µM. PCRs were carried out in 96-well plates using the CFX Connect Real-Time PCR Detection System (Bio-Rad, Marnes-la-Coquette, France) with the following temperature cycling: 95 °C for 30 s followed by 40 cycles consisting of 95 °C for 5 s followed by 60 °C for 30 s and finally a melt curve analysis from 65 °C to 95 °C to detect unspecific PCR products. All steps were done following the manufacturer’s instructions. Statistical analyses for RT-q-PCR were done using the CFX Maestro software. The forward and reverse primers for reverse transcription-quantitative polymerase chain reaction (RT-qPCR) of *MMACHC*, *TESK2* transcripts, and *PRDX1* aberrant transcript are reported in Additional file 3: Table S1.

## Results

### Epigenome-wide association study for the epi-*cblC* phenotype

**Fig. 1:**
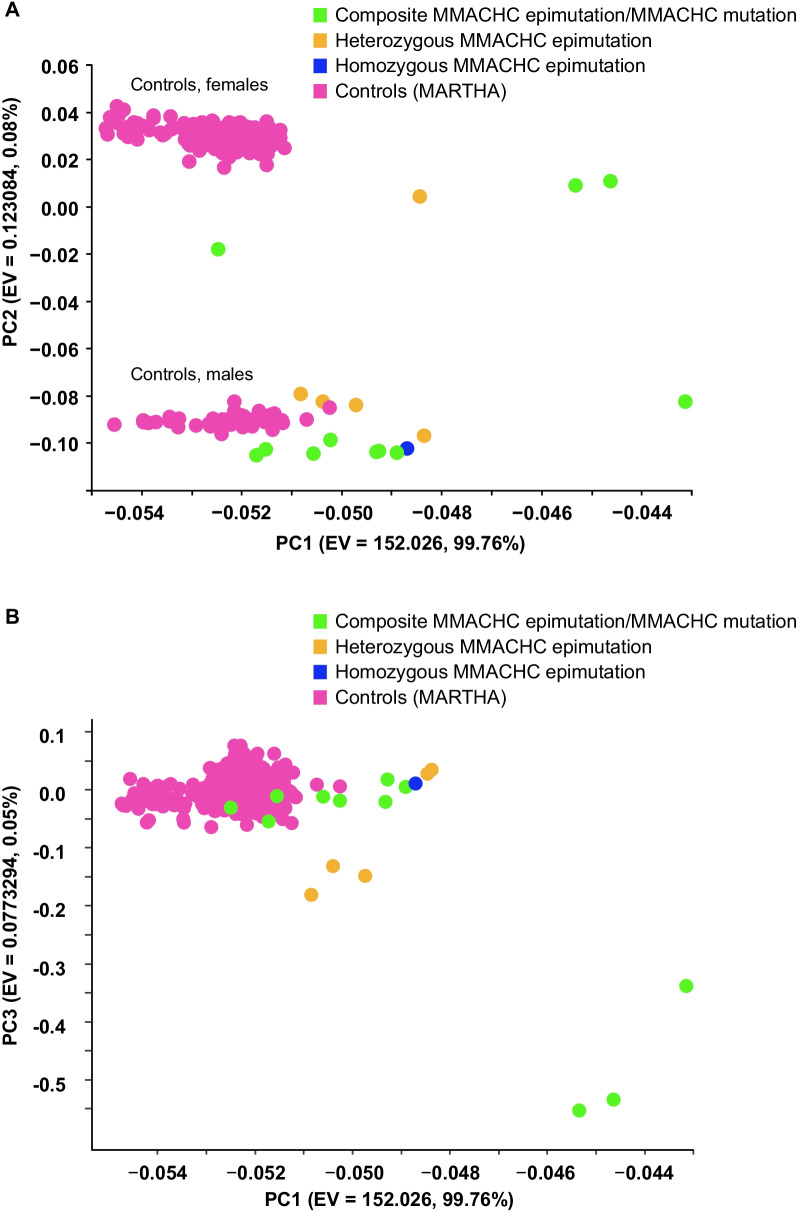
**A** 2-D plot using the two top eigenvectors (PC1, PC2) derived from the primary component analysis on the genome-wide methylome landscape of the studied patients and controls. **B** 2-D plot using the PC1 and PC3 eigenvectors derived from the primary component analysis on the genome-wide methylome landscape of the studied patients and controls

**Fig. 2:**
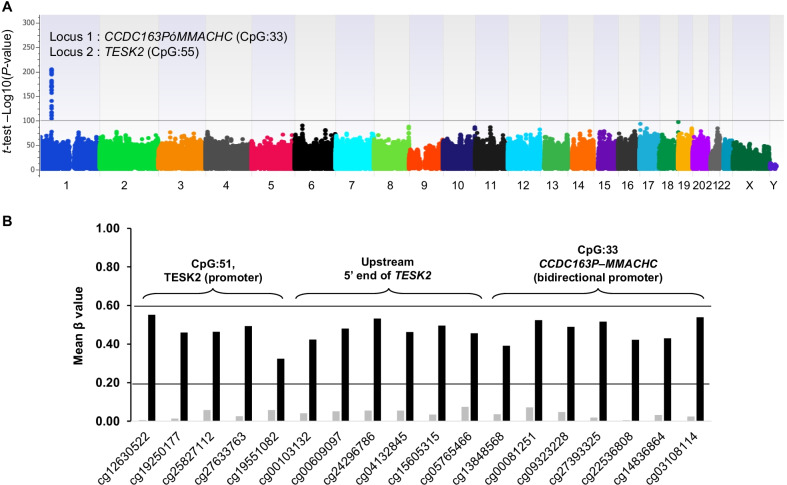
**A** Epi-Manhattan plot reporting the results of the epigenome-wide association study that compared 17 patients with *MMACHC* epimutation (epi-*cblC* disease, isolated *MMACHC* epimutation, biallelic *MMACHC* epimutation) with controls. The − log10 *P*-value reports the *t*-test that compared the mean β values between the two groups. The horizontal line indicates a *P*-value threshold of 1 × 10^−100^. The top significant hit in chromosome 1 corresponds to the CpG island (CpG:33) on the bidirectional promoter of *CCDC163*–*MMACHC* and the CpG island (CpG:51) on the promoter region of the *TESK2* gene. **B** Epigrams reporting the methylation levels of the top CpG probes from the epigenome-wide association study results that compared 17 patients with *MMACHC* epimutation (epi-*cblC* disease, isolated *MMACHC* epimutation, biallelic *MMACHC* epimutation) (black bars) with controls (gray bars). The horizontal lines correspond to β value thresholds of 0.2, below which the CpG probe is considered to be fully unmethylated. Above 0.6, the CpG probe is considered fully methylated. A β value between 0.2 and 0.6 indicates a hemimethylated CpG probe. All the CpG probes located in CpG islands CpG:33 (*CCDC163P*–*MMACHC* bidirectional promoter), upstream the 5’ end of the *TESK2* promoter, and the CpG:51 (*TESK2* promoter) were fully unmethylated among controls and exhibited a hemimethylated profile among cases

**Fig. 3:**
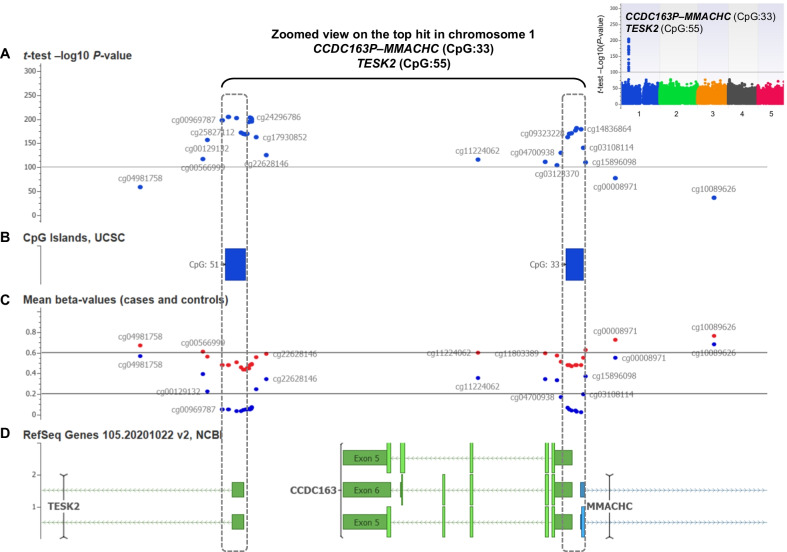
**A** Zoomed view of the epi-Manhattan plot reporting the epigenome-wide association study results that compared 17 patients with *MMACHC* epimutation (epi-*cblC* disease, isolated *MMACHC* epimutation, biallelic *MMACHC* epimutation) with controls. The − log10 *P*-value reports the *t*-test that compared the mean β values between the two groups. The horizontal line indicates a *P*-value threshold of 1 × 10^−100^. The zoomed view of the top epigenomic signature in chromosome 1 confirmed the significant association at the CpG island ‘CpG:33’ on the *CCDC163*–*MMACHC* bidirectional promoter but also revealed a second significant association at the CpG island ‘CpG:51’ on the promoter region of the *TESK2* gene. **B** Positions of the CpG islands CpG:33 and CpG:51 according to the CpG Islands UCSC annotation. **C** Mean β values among the 17 patients with *MMACHC* epimutation (epi-*cblC* disease, isolated *MMACHC* epimutation, biallelic *MMACHC* epimutation) (red dots) and controls (blue dots). The horizontal lines correspond to β value thresholds of 0.2, below which the CpG probe is considered to be fully unmethylated. Above 0.6, the CpG probe is considered fully methylated. A β value between 0.2 and 0.6 indicates a hemimethylated CpG probe. **D** Genomic annotation according to RefSeq Genes 105.20201022 v2, NCBI

All the DNA methylome profiles passed the quality checks and exhibited a valid β value density distribution (Additional file 1: Figure S1). In PCA on genome-wide DNA methylome profiles, we found a clustered distribution according to patients’ gender (EV2) and the epi-*cblC* phenotype (EV1) (Fig. 1). Additionally, we performed a PCA analysis that only considered CpG probes on chromosome 1. The PCA plot reporting PC1 vs. PC2 from chromosome 1-based PCA analysis was similar to that obtained from the genome-wide PCA analysis (PC1 vs. PC3) (Additional file 1: Figure S2). The EWAS revealed a significant locus in chromosome 1 in association with the epi-*cblC* phenotype (Fig. 2A). The visualization of this top epigenomic signature confirmed the significant association at the CpG island ‘CpG:33’ on the *CCDC163*–*MMACHC* bidirectional promoter but also revealed a second significant association at the CpG island ‘CpG:51’ on the promoter region of the *TESK2* gene (Figs. 2B and 3). The mean-beta values of the CpG probes located in the CpG islands CpG:33 and CpG:51 had a fully unmethylated profile among controls and a hemimethylated profile among cases (Table 1 and Figs. 2B and 3).

**Table 1:** Top significant CpG probe in the epigenome-wide association study on the epi-*cblC* phenotype

CpG probe	Chr	Position*	CpG island	Locus	*t*-test, *P*-value	*t*-test, Bonf. *P*-value	β-value (Cases)	β-value (Controls)	Δβ-value
cg12630522	1	45956424	CpG:51	*TESK2 (promoter)*	3.86E-223	1.74E-217	0.55	0.01	0.54
cg19250177	1	45956646	CpG:51	*TESK2 (promoter)*	7.56E-204	3.42E-198	0.46	0.01	0.45
cg25827112	1	45956773	CpG:51	*TESK2 (promoter)*	4.96E-192	2.25E-186	0.46	0.06	0.41
cg27633763	1	45956828	CpG:51	*TESK2 (promoter)*	1.22E-94	5.53E-89	0.49	0.03	0.47
cg19551082	1	45956882	CpG:51	*TESK2 (promoter)*	3.62E-148	1.64E-142	0.32	0.06	0.27
cg00103132	1	45956932	Upstream 5’ end CpG:51	Upstream 5’ end *TESK2*	4.42E-212	2.00E-206	0.42	0.04	0.38
cg00609097	1	45956974	Upstream 5’ end CpG:51	Upstream 5’ end *TESK2*	6.61E-195	2.99E-189	0.48	0.05	0.43
cg24296786	1	45957014	Upstream 5’ end CpG:51	Upstream 5’ end *TESK2*	2.75E-199	1.24E-193	0.53	0.05	0.48
cg04132845	1	45957038	Upstream 5’ end CpG:51	Upstream 5’ end *TESK2*	1.96E-218	8.85E-213	0.46	0.06	0.41
cg15605315	1	45957053	Upstream 5’ end CpG:51	Upstream 5’ end *TESK2*	3.07E-196	1.39E-190	0.50	0.03	0.46
cg05765466	1	45957060	Upstream 5’ end CpG:51	Upstream 5’ end *TESK2*	8.34E-203	3.77E-197	0.46	0.08	0.38
cg13848568	1	45965625	CpG:33	*CCDC163P–MMACHC* (bidirectional promoter)	1.87E-138	8.44E-133	0.39	0.04	0.35
cg00081251	1	45965679	CpG:33	*CCDC163P–MMACHC* (bidirectional promoter)	2.89E-182	1.31E-176	0.52	0.07	0.45
cg09323228	1	45965727	CpG:33	*CCDC163P–MMACHC* (bidirectional promoter)	1.18E-171	5.33E-166	0.49	0.05	0.44
cg27393325	1	45965846	CpG:33	*CCDC163P–MMACHC* (bidirectional promoter)	2.16E-195	9.79E-190	0.52	0.02	0.50
cg22536808	1	45965870	CpG:33	*CCDC163P–MMACHC* (bidirectional promoter)	7.73E-167	3.50E-161	0.42	0.00	0.42
cg14836864	1	45965990	CpG:33	*CCDC163P–MMACHC* (bidirectional promoter)	2.33E-167	1.05E-161	0.43	0.03	0.40
cg03108114	1	45966048	CpG:33	*CCDC163P–MMACHC* (bidirectional promoter)	1.51E-208	6.82E-203	0.54	0.02	0.51

*Chr* chromosome; *Bonf.* Bonferroni; *Δβ-value* difference between case and control β values

^*^Genomic positions are reported according to GRCh37

### Genome-wide chromatin landscape profiling in fibroblasts of patients with epi-*cblC*

**Fig. 4:**
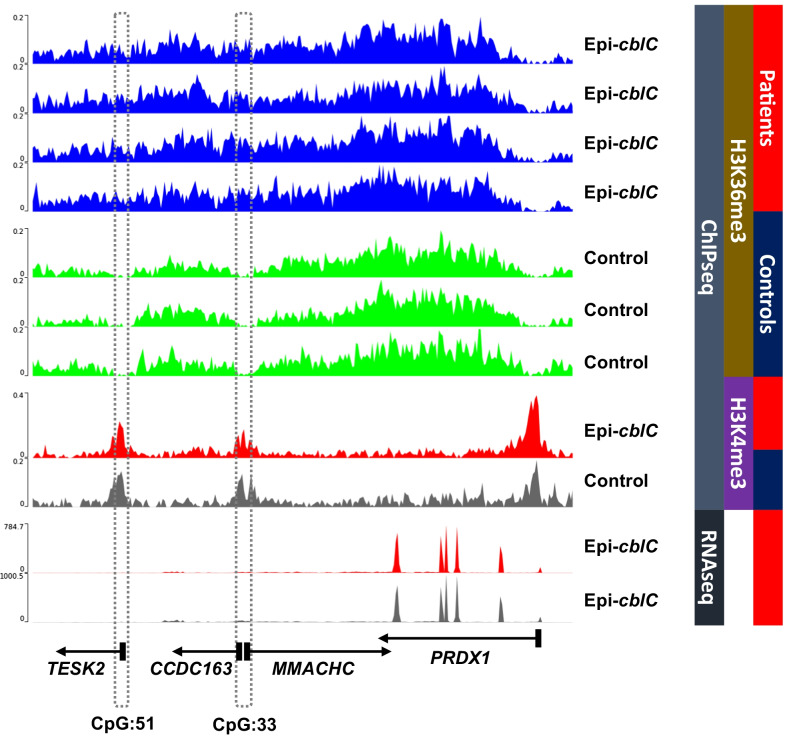
Results of ChIP-Seq analyses at the genomic region encompassing the *CCDC163*–*MMACHC* bidirectional promoter region (CpG:33) and in the *TESK2* promoter region (CpG:51). Genomic panels show normalized coverage for histone H3 trimethylated lysine 36 (H3K36me3) and H3K4me3 marks in patients with an epi-*cblC* phenotype and controls. The dashed rectangles indicate the *CCDC163–MMACHC* bidirectional promoter region (CpG:33) and the *TESK2* promoter region (CpG:51). The same scale has been set in all panels. ChIP-Seq showed a significant accumulation of the H3K36me3 and a mirrored profile of H3K4me3 in the *CCDC163–MMACHC* bidirectional promoter region (CpG:33) and in the *TESK2* promoter region (CpG:51) among patients with an epi-*cblC* phenotype in comparison with controls. In contrast, the histone H3K36me3 and H3K4me3 marks are similar in the *PRDX1* promoter region. The RNAseq showed a high expression of *PRDX1* exons and low or no expression of *MMACHC* exons in case WG-3838 epi-*cblC* and *cblC* fibroblasts

We assessed ChIP-Seq data generated on case and control fibroblasts previously described in reference 2 to further characterize the epigenetic changes at the *MMACHC* and *TESK2* loci. We observed a significant accumulation of trimethylated lysine 36 on histone H3 (H3K36me3) and no mark of H3K4me3 in the *CCDC163*–*MMACHC* bidirectional promoter region (CpG:33) and in the *TESK2* promoter region (CpG:51) among patients with an epi-*cblC* phenotype in comparison with controls (Fig. 4). Overall, the region spanning from mid-*PRDX1* to *TESK2* harbored a continuous enrichment of H3K36me3 marks in epi-*cblC* patients as compared to controls (Fig. 4).

### The epimutations in *CCDC163*–*MMACHC* bidirectional promoter and *TESK2* promoter result from *PRDX1* antisense transcription

**Fig. 5:**
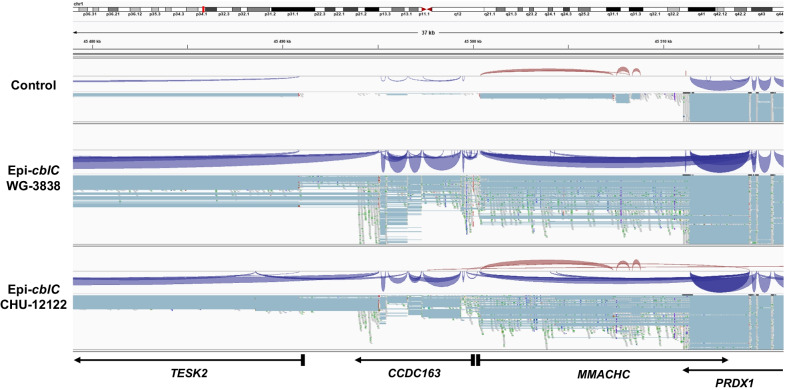
Overview of the transcription anomalies with aberrant splicing and wrong splice junction usage in the genomic region encompassing *TESK2*, *CCDC163*, *MMACHC,* and *PRDX1*. For every three samples, two tracks are represented: i) a sashimi plot with splice junction usage (thickness of the line is proportional to read depth supporting the splice junctions; in red: forward strand processing; in blue: reverse strand processing), and ii) an exhaustive read coverage in squished mode, with horizontal blue-grey lines indicating inserts between paired-end reads; In this view, well-defined horizontal gaps represent exons when introns are successfully spliced. Alignment was made with HISAT2 on the reference genome hg38

**Fig. 6:**
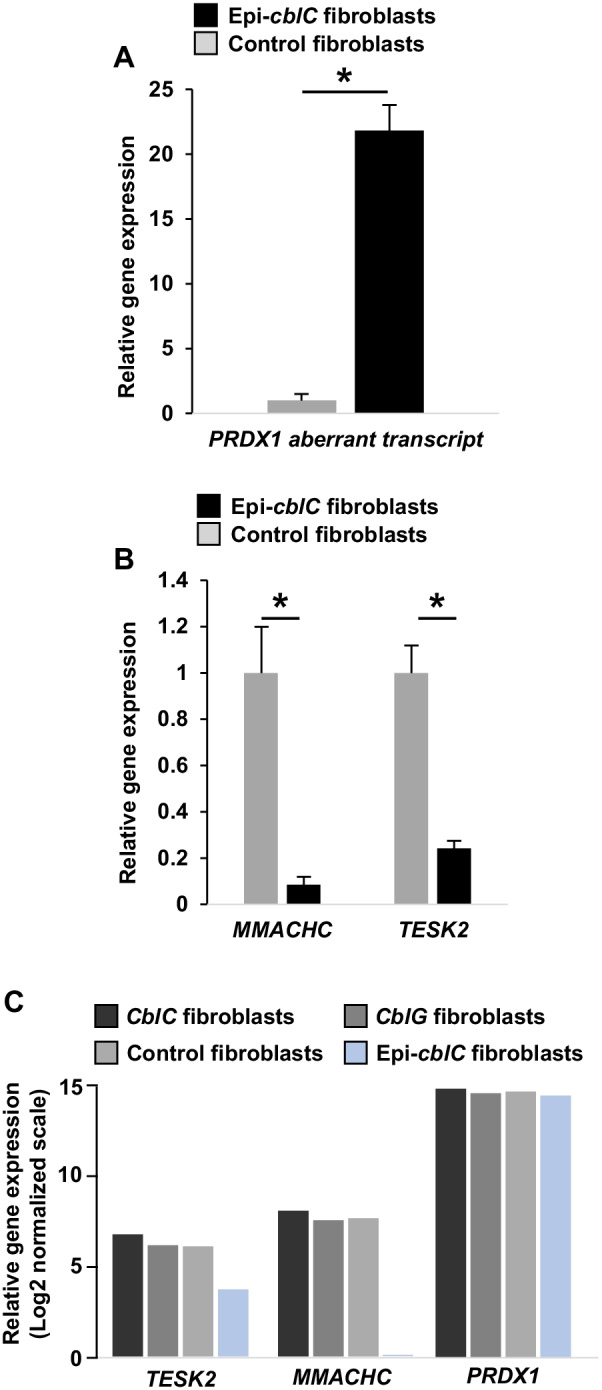
**A** Relative expression levels of *PRDX1* aberrant transcript in CHU-12122 epi-*cblC* fibroblasts to control fibroblasts normalized to *GAPDH* and *TBP*; **B** relative expression levels of *MMACHC* and *TESK2* mRNAs in epi-*cblC* fibroblasts relative to control fibroblasts normalized to *GAPDH* and *TBP* (*P < 0.001, *n* = 5 analysed cell flasks). **C** Expression levels of canonical transcripts for *TESK2*, *MMACHC* and *PRDX1* genes in RNA-seq analyses of fibroblasts from epi*-cblC* (*n* = 2), *cblC* (*n* = 3) and *cblG* (*n* = 3) cases and control fibroblasts (*n* = 4). Expressions in log scale

The RNA-seq analyses showed aberrant splicing and wrong splice junction usage in the genomic region encompassing *TESK2*, *CCDC163*, *MMACHC,* and *PRDX1* (Fig. 5). The RNA-seq analyses showed the absence of *MMCHC* sense transcripts in case WG-3838, a result which may be consistent with a splicing defect produced by the c.80A > G variant in *MMACHC*, which may then lead to total degradation of the mRNA (Fig. 5). In contrast, in case CHU-12122, we detected transcripts with the c.270_271insA mutation (heterozygous c.270_271insA, p.Arg91LysfsTer14) (Fig. 5). The RNA-Seq and RT-qPCR analyses of case fibroblasts showed a *PRDX1* readthrough transcription through the *CCDC163*–*MMACHC* bidirectional promoter region (CpG:33) but also through the *TESK2* promoter region (CpG:51) (Figs. 5 and 6). In epi-*cblC* fibroblasts, after de novo transcriptome reconstruction in the region spanning from *PRDX1* to *TESK2*, we observed a dramatic increase in the *PRDX1* aberrant transcripts as compared with control fibroblasts (Fig. 5). We further assessed the expression of *MMACHC*, *TESK2*, and *PRDX1* aberrant transcripts in fibroblasts of patients with composite epi-*cblC*. In comparison with controls, fibroblasts from the CHU-12122 patient with epi-*cblC* showed a 22-fold increase of *PRDX1* aberrant transcript and a dramatic decrease of both *MMACHC* (12-fold decrease) and *TESK2* (fourfold decrease) mRNA, in RT-qPCR analyses normalized to *GAPDH* and *TBP* (Fig. 6B). The canonical transcriptions of *TESK2* and *MMACHC*, but not *PRDX1,* were also decreased in the RNA-seq analysis of CHU-12122 and WG-3838 fibroblasts (Fig. 6C). In contrast, the canonical transcription of *MMACHC* and *TESK2* was similar to that of *PRDX1* in RNA-seq and RT-qPCR analysis of control cells (Fig. 6C).

## Discussion

We found that the two *PRDX1* mutations reported in 17 epi-*cblC* cases produced antisense readthrough transcripts encompassing the *CCDC163P*/*MMACHC* bidirectional promoter and the *TESK2* promoter, resulting in H3K36me3 marks, generation of epimutations in the two CpG islands and silencing of *MMACHC* and *TESK2* genes. These results showed that epi-*cblC* is a clinical entity that must be distinguished from *cblC* with regard to the potential consequences that may be produced by *TESK2* silencing. Epi-*cblC* is an example of an altered expression of two genes through a common epigenetic mechanism, which led us to introduce the term 'epi-digenism.'

The previous reports of the clinical and metabolic presentations of the 17 epi-*cblC* cases showed similar manifestations, compared to the *cblC* type of inherited disorders of cobalamin metabolism, with combined methylmalonic aciduria and homocystinuria, hypotonia, failure to thrive, megaloblastic anemia, pulmonary hypertension, and fatal neonatal cardio-metabolic decompensation [2, 3, 16–18]. Of note, the clinical phenotype of the epi-*cblC* homozygote appeared to be more severe than homozygous cases with *MMACHC* c.271dupA, which is the most common mutation reported in European patients with *cblC* [3, 18]. In addition, some manifestations are frequent in epi-*cblC*, including skeletal deformity, metabolic acidosis with or without hyperammonemia, and recurrent severe infections [3]. The presence of the *PRDX1* mutation and the potential clinical consequences of *TESK2* silencing in epi-*cblC* cases suggest that it should be considered as a distinct entity. *PRDX1* encodes Peroxiredoxin 1, a versatile protein involved in cell defense against cellular oxidative stress, influencing cell growth, differentiation, and apoptosis [19]. PRDX1 enhances the natural killer cell cytotoxicity [20] and inhibits the function of oncoproteins such as c-Abl [21] and c-Myc [19]. The role of *PRDX1* in suppressing tumors has been confirmed in *Prdx1*-knockout mouse models [22, 23]. We suggest periodically monitoring subjects bearing the *c.515-1G* > *T* variant to test the hypothesis of a potential increased risk of cancer. This potential risk could be related to the decreased expression of *PRDX1* evidenced in RNA-seq analyses (Fig. 5). The *PRDX1*:c.515-1G > T variant leads to the skipping of the last exon, which encodes one of the two cysteine residues which are essential for the catalytic activity [2, 3]. We also observed a clear reduction of the expression of *TESK2*, which could impact the disease course of epi-*cblC* cases. *TESK2* encodes a putative 555-amino acid protein with a kinase domain that exhibits a dual specific protein kinase activity on both serine/threonine and tyrosine residues [24]. The low expression of *TESK2* is associated with poor survival of patients with lung adenocarcinoma and premetastatic stage in human lung-to-brain metastasis [25]. *TESK2* is mainly expressed in the testis and prostate and influences cofilin phosphorylation and actin scaffold in testicular Sertoli cells [24, 26]. It also influences myogenic differentiation through actin remodeling [27]. *CCDC163P* was also silenced and was predicted to be the coding gene of a transmembrane protein, which has a main transcriptional expression in the thyroid, brain, and testis [28]. However, the protein has not been characterized and it is not associated with pathological outcomes [28].

The RNA-seq data analysis showed that the readthrough transcription of *PRDX1* was prolongated through the *MMACHC*/*CCDC163P* bidirectional promoter and the *TESK2* promoter in fibroblasts of epi-*cblC* patients (Fig. 5). The H3K36me3 mark was the predominant common characteristic of histone marks at both *MMACHC* and *TESK2* promoters (Fig. 4). This mark is deposited only by the histone-lysine N-methyltransferase SETD2 at K36 of histone H3, where it binds the active form of RNA polymerase II [29]. Subsequently, the H3K36me3 mark allows the recruitment of DNMT3B1 through the binding of the heterochromatin protein 1 (HP1). This explains why the de novo methylation of CpG islands is preferentially targeted to genomic regions with elevated H3K36me3 levels through the recruitment of DNMT3B1 in mouse stem cells [30]. Based on these data and our data on RNA-seq and ChIP-seq of patient fibroblasts, we propose a common mechanism that produces the writing of the two epimutations through the successive recruitment of SETD2, HP1, and DNMT2B in epi-*cblC* disease. The RNA-seq analyses showed the activation of numerous cryptic splice sites that participated in the readthrough transcription of *PRDX1* and its prolongation through the *MMACHC*, *CCDC163P,* and *TESK2* genes. The activation of these cryptic splice sites could involve SETD2. Indeed the SETD2 methyltransferase not only mediates the co-transcriptional methylation in histone H3 but it also has other functions that include the regulation of pre-mRNA splicing [31].

Digenic inheritance (DI) concerns pathologies with multigenic etiology implicating more than one gene [32]. Examples of unequivocal digenism in known diseases have been reviewed previously [32]. One of the categories of digenism is the inheritance of a single primary mutation that establishes the diagnosis and a second DNA variant, which modifies the phenotype [32]. In the case of epi-*cblC*, the epimutation in the *CCDC163P/MMACHC* promoter produces the main phenotype of the disease while the epimutation in the promoter of *TESK2* produces its decreased expression with potential clinical consequences [25]. Therefore, we propose introducing the term ‘epi-digenism’ and considering epi-*cblC* as an example of epidigenism since the silencing of *MMACHC* and *TESK2* are produced by a shared epigenetic mechanism on the two promoters.

Recently, a comprehensive genome-wide analysis of human epivariations analyzed in silico data generated from 23,116 individuals with the Illumina 450 k methylation array and reported 4,452 unique autosomal epivariations, including 384 in disease-causing OMIM genes [33]. The epivariation in the *CCDC163P*/*MMACHC* promoter was the most frequent among the 384 hypermethylated in the studied individuals [17]. Of note, one of the subjects had epivariations in *TESK2* and *CCDC163P*/*MMACHC* promoters [17]. This suggests that both epivariations could be triggered by environmental factors, as reviewed recently for *CCDC163P*/*MMACHC* promoter [17]. In addition, several EWAS from the EWAS catalog (http://ewascatalog.org/?cpg=cg0036120) showed the association of hypermethylated CpG in *CCDC163P*/*MMACHC* and *TESK2* promoters with sex, age, child abuse, rheumatoid arthritis, and five-year Increase in epigenomic biological age of subjects with chronic HIV Infection [34–37].

In conclusion, our results show that epi-*cblC* should be distinguished from *cblC*, with potential consequences on spermatogenesis and cancer risk produced by *TESK2* silencing. The coverage of readthrough transcripts through a large region that encompasses *MMACHC*, *CCDC163P* and *TESK2* involves the activation of numerous splice cryptic sites. Epi-*cblC* is an example of altered expression of two neighboring genes through a shared epigenetic pathomechanism. We propose introducing the term 'epi-digenism' to characterize this type of disorder.

## Supplementary Information

**Additional file 1. Supplemental Figure S1.** Genome-wide density distribution of CpG probes in the 17 DNA methylome profiles of the 17 patients with *MMACHC* epimutation (epi-*cblC* disease, isolated *MMACHC* epimutation, biallelic *MMACHC* epimutation).

**Additional file 2. Supplemental Figure S2.** (**A**) 2-D plot using the two top eigenvectors (PC1, PC3) derived from the primary component analysis on the genome-wide methylome landscape of the studied patients and controls. (**B**) 2-D plot using the PC1 and PC2 eigenvectors derived from the primary component analysis on the methylome landscape of chromosome 1 of the studied patients and controls.

**Additional file 3. Supplemental Table S1.** Forward and reverse primers for reverse transcription-quantitative polymerase chain reaction (RT-qPCR) of MMACHC, TESK2 transcripts and PRDX1 aberrant transcript.

## Data Availability

Data are available for use in collaborative studies to researchers upon reasonable request (jean-louis.gueant@univ-lorraine.fr). Data will be provided following the review and approval of a research proposal (including a statistical analysis plan) and the completion of a data-sharing agreement. Responses to the request for the raw data will be judged by the IRB of INSERM UMR_S 1256.
