# Supplementary figures and images for: Epimutations in both the TESK2 and MMACHC promoters in the Epi-cblC inherited disorder of intracellular metabolism of vitamin B12
Source: Clin Epigenetics. 2022 Apr 19;14:52. doi: 10.1186/s13148-022-01271-1 (PMC9020039; doi:10.1186/s13148-022-01271-1)

## Slide 1
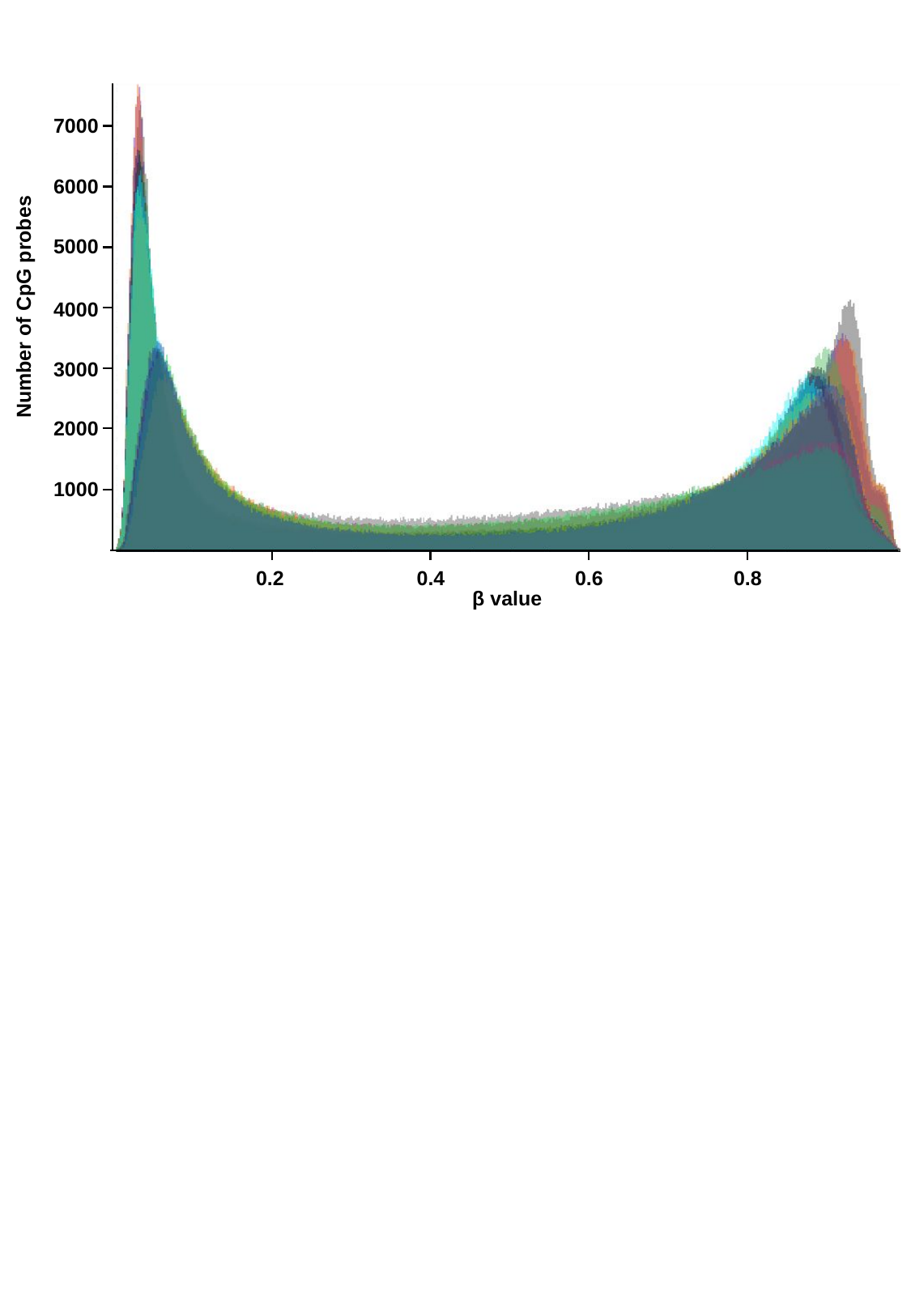

7000
6000
5000
Number of CpG probes
4000
3000
2000
1000
0.2
0.4
0.6
0.8
β value

Supplement: Supplementary file 1 — Additional file 1. Supplemental Figure S1. Genome-wide density distribution of CpG probes in the 17 DNA methylome profiles of the 17 patients with MMACHC epimutation (epi-cblC disease, isolated MMACHC epimutation, biallelic MMACHC epimutation). [file 13148_2022_1271_MOESM1_ESM.pptx]
