# Supplementary material for: Epimutations in both the TESK2 and MMACHC promoters in the Epi-cblC inherited disorder of intracellular metabolism of vitamin B12
Source: Clin Epigenetics. 2022 Apr 19;14:52. doi: 10.1186/s13148-022-01271-1 (PMC9020039; doi:10.1186/s13148-022-01271-1)
Supplement: Supplementary file 2 — Additional file 2. Supplemental Figure S2. (A) 2-D plot using the two top eigenvectors (PC1, PC3) derived from the primary component analysis on the genome-wide methylome landscape of the studied patients and controls. (B) 2-D plot using the PC1 and PC2 eigenvectors derived from the primary component analysis on the methylome landscape of chromosome 1 of the studied patients and controls. [file 13148_2022_1271_MOESM2_ESM.pptx]

## Slide 1
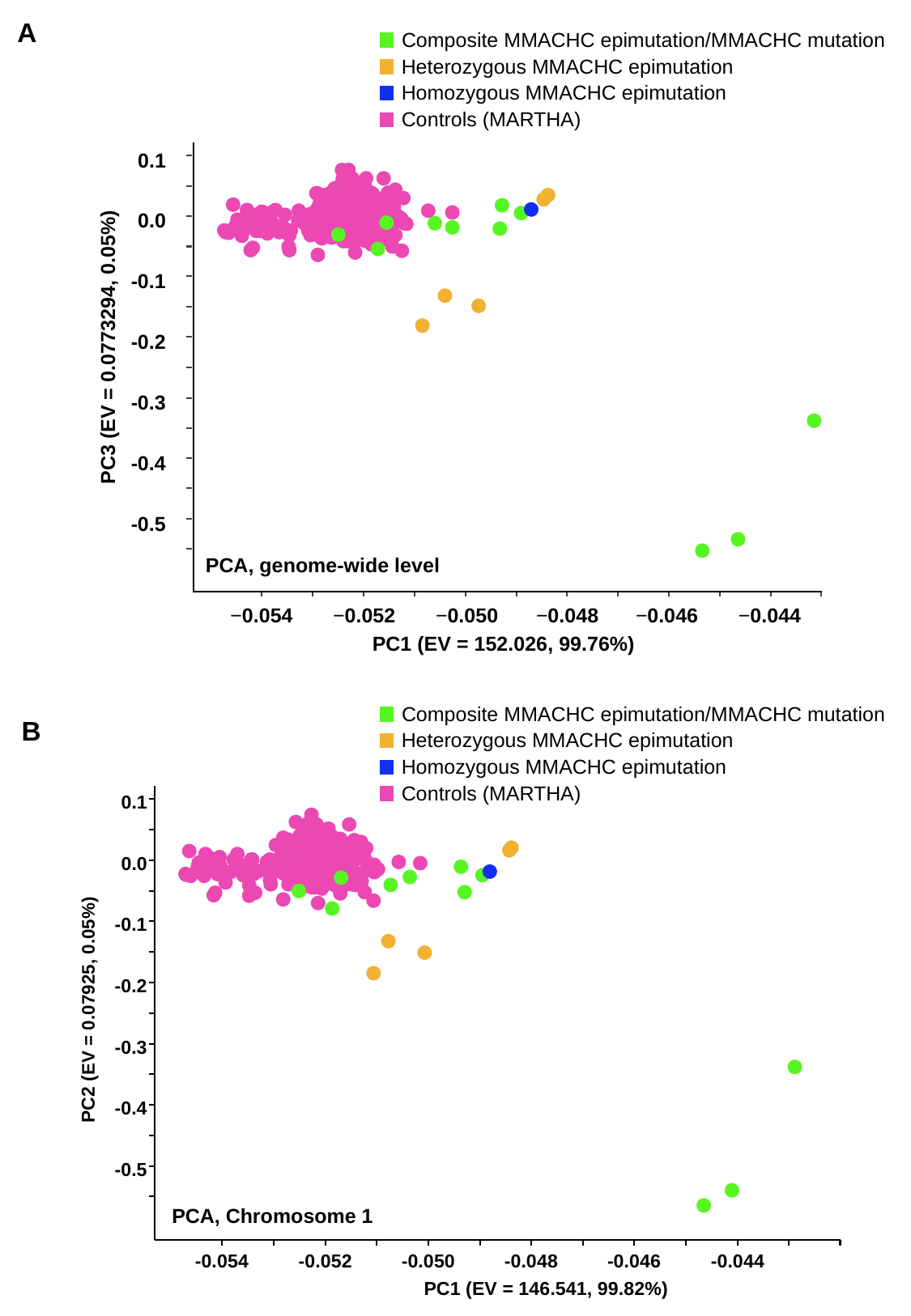

A
Composite MMACHC epimutation/MMACHC mutation
Heterozygous MMACHC epimutation
Homozygous MMACHC epimutation
Controls (MARTHA)
0.1
0.0
-0.1
-0.2
PC3 (EV = 0.0773294, 0.05%)
-0.3
-0.4
-0.5
−0.054
−0.052
−0.050
−0.048
−0.046
−0.044
PC1 (EV = 152.026, 99.76%)
Composite MMACHC epimutation/MMACHC mutation
Heterozygous MMACHC epimutation
Homozygous MMACHC epimutation
Controls (MARTHA)
B
0.1
0.0
-0.1
-0.2
PC2 (EV = 0.07925, 0.05%)
-0.3
-0.4
-0.5
-0.054
-0.052
-0.050
-0.048
-0.046
-0.044
PC1 (EV = 146.541, 99.82%)
PCA, genome-wide level
PCA, Chromosome 1
