# Supplementary material for: Epimutations in both the TESK2 and MMACHC promoters in the Epi-cblC inherited disorder of intracellular metabolism of vitamin B12
Source: Clin Epigenetics. 2022 Apr 19;14:52. doi: 10.1186/s13148-022-01271-1 (PMC9020039; doi:10.1186/s13148-022-01271-1)
Supplement: Supplementary file 3 — Additional file 3. Supplemental Table S1. Forward and reverse primers for reverse transcription-quantitative polymerase chain reaction (RT-qPCR) of MMACHC, TESK2 transcripts and PRDX1 aberrant transcript. [file 13148_2022_1271_MOESM3_ESM.docx]

**Supplemental Table S1. Forward and reverse primers for reverse transcription-quantitative polymerase chain reaction (RT-qPCR) of *MMACHC*, *TESK2* transcripts and *PRDX1* aberrant transcript.**

|  | **Forward primer (5’-3’)*** | **Reverse primer (5’-3’)*** |
| --- | --- | --- |
| ***PRDX1* aberrant**  **transcript** | ACCATATTGCCTATGCGCGA | GAGGCGGTGGAACTACCTTT |
| ***MMACHC*** | ACGGCCCAATTGTCCTTGAG | GGCTGGAGGCAAGAGTTCATT |
| ***TESK2*** | AACCTGCATTTGCCTTGGAC | GCAGAGTAACCATTCTCATCCCTC |
| ***TBP*** | CGAACCACGGCACTGATTTTC | TCACAGCTCCCCACCATATTC |
| ***GAPDH*** | TGTTCGTCATGGGTGTGA | AGGGATGATGTTCTGGAGA |

*Primers were designed using Primer-BLAST.
